# Steam explosion pretreatment enhancing enzymatic digestibility of overground tubers of tiger nut (*Cyperus esculentus* L.)

**DOI:** 10.3389/fnut.2022.1093277

**Published:** 2023-01-06

**Authors:** Zhi-Min Zhao, Wenqing Yu, Caitong Huang, Huiting Xue, Juan Li, Dejian Zhang, Guanhua Li

**Affiliations:** ^1^State Key Laboratory of Reproductive Regulation and Breeding of Grassland Livestock, School of Life Sciences, Inner Mongolia University, Hohhot, China; ^2^Inner Mongolia Key Laboratory of Environmental Pollution Control & Wastes Reuse, School of Ecology and Environment, Inner Mongolia University, Hohhot, China; ^3^College of Basic Medicine, Inner Mongolia Medical University, Hohhot, China

**Keywords:** lignocellulosic biomass, tiger nut, enzymatic hydrolysis, steam explosion pretreatment, biorefinery

## Abstract

**Introduction:**

Tiger nut (TN) is recognized as a high potential plant which can grow in well-drained sandy or loamy soils and provide food nutrients. However, the overground tubers of TN remain unutilized currently, which limits the value-added utilization and large-area cultivation of this plant.

**Methods:**

In the present study, the overground tubers of TN were subjected to enzymatic hydrolysis to produce fermentable sugars for biofuels production. Steam explosion (SE) was applied to modify the physical-chemical properties of the overground tubers of TN for enhancing its saccharification.

**Results and discussion:**

Results showed that SE broke the linkages of hemicellulose and lignin in the TN substrates and increased cellulose content through removal of hemicellulose. Meanwhile, SE cleaved inner linkages within cellulose molecules, reducing the degree of polymerization by 32.13–77.84%. Cellulose accessibility was significantly improved after SE, which was revealed visibly by the confocal laser scanning microscopy imaging techniques. As a result, enzymatic digestibility of the overground tubers of TN was dramatically enhanced. The cellulose conversion of the SE treated TN substrates reached 38.18–63.97%, which was 2.5–4.2 times higher than that without a SE treatment.

**Conclusion:**

Therefore, SE pretreatment promoted saccharification of the overground tubers of TN, which paves the way for value-added valorization of the TN plants.

## Introduction

Energy security is essential for the development of global economy. Traditional fossil fuels (e.g., oil, coal) are still the main energy sources in the world currently. However, the consumption of fossil fuels leads to severe environmental deterioration (1). Broad consensus on fossil fuel reduction and pollution minimization stipulates great research interest to renewable fuels (2). Nearly all cellulose is produced by photosynthetic higher plants and algae in the nature, and is considered to participate in the most vital global carbon flows. Additionally, cellulose is the only origin of “green” fixed carbon source and widely available worldwide (3). Biofuels from inedible cellulose are deemed as clean and renewable alternatives to the traditional fossil fuels, which mitigate climate change and reduce greenhouse gas emission. Commercial biofuels require cheap resources that are easy to harvest and collect. Tiger nut (*Cyperus esculentus* L., TN), a *Corsa sedge* genus perennial herb, is dated back to ~4,000 years ago and now widely cultivated in the tropic or temperate regions, with an annual mass of 9,000 metric tons (4). TN can be a major grower in well-drained sandy or loamy soils to achieve a high productivity and economic efficiency. The underground tubers of TN contain unsaturated fatty acids, protein, and vitamin E, which are commonly regarded as a “health” food. However, there is seldom report regarding the utilization of the overground tubers, despite more than 90 cm in height of the solitary stems growing from a tuber. Overground tubers of TN represent promising resources for biofuels production.

The key step in biofuels production is to release fermentable sugars form cellulose with economic competitiveness (5). Compared to chemical strategies, biological conversion of cellulose exhibits more attractiveness owing to the lower energy input and less environment pollution (6). Enzymatic hydrolysis of cellulose needs valid binding between the surface of substrate and cellulase. Cellulose is a linear polymer condensed by D-anhydroglucopyranose through β-1,4-glycosidic bonds, which exists as successive and stacked sheets of anhydroglucopyranose on top of each other, forming a three-dimensional particle. This particle exhibits distinct “faces” that interact with the aqueous environment and enzymes (7). The “faces” can be deemed as both external and internal surfaces. The former is closely related to crystallinity index (CrI), degree of polymerization (DP), and pore size, whereas the latter is correlated with shape or particle size. For example, it is reported that crystalline cellulose hydrolysis rates are commonly 3–30 times faster than amorphous cellulose (8). Additionally, other components, such as hemicellulose, lignin, and pectin, are commonly interconnected through non-covalent and covalent linkages, forming a complicated structural and chemical network coats the “faces” or generates non-productive enzyme binding. This physical and chemical complexity makes cellulose recalcitrant to enzymatic hydrolysis. Additionally, the recalcitrance presents substantial differences according to different biomass, organs, and maturity stages. Therefore, establishing appropriate pretreatment and saccharification technologies to improve the cellulose accessibility to cellulases is significantly important for enhancing the enzymatic digestibility.

Pretreatment is considered to be a foundational step to efficiently facilitate enzymatic hydrolysis through removing stubborn components and deconstructing lignocellulosic matrix, which is also a key criterion to determine economic viability of large-scale industrialization (9). Steam explosion (SE), a kind of mechanico-physico-chemical pretreatment, is becoming one of the most commonly used technologies with huge commercial potential, because it needs low capital investment and energy requirements (10). Also, SE is a clean process without any chemical addition. During the SE process, the sample is treated by saturated steam at high temperature and pressure for a short duration time (cooking stage), during which the hydrolytic breakdown occurs. And then a sudden pressure drop (decompression phase) occurs, which results in a vapor expansion inside the “capillary-like” structures and a dislocation of the fibrous structure. SE has been proven efficient for fractionating a broad range of lignocellulosic feedstock, including wheat straw, sugarcane bagasse, and corn stover. The byproducts could be generated during the SE process, which could be toxic for the following valorization processes. These inhibiting compounds are divided into three main groups: weak acids, furan derivatives, and phenolic compounds (11). In detail, acetic acid is formed from hemicellulose, which further reduces the pH of the cooking liquor. The low pH and high temperature during SE could promote monosaccharide degradation to generate furfural, 5-hydroxymethylfurfural (HMF), and levulinic acid. The phenolic compounds are formed from lignin degradation (12). Our previous studies have shown that the redistribution of lignin facilitates the enzymatic digestibility of cellulose (13). However, the precise mechanism from an integral sense, especially, the influence of lignin distribution around the surface of cell wall caused by SE pretreatment on the cellulose accessibility remains ambiguous. To the best of our knowledge, SE pretreatment of TN biomass is seldom studied. In order to facilitate the value-added utilization of the TN plants, SE was applied to modify the physical-chemical properties of the overground tubers of TN. The chemical compositions and physical parameters (e.g., CrI and DP) were measured to investigate the influences of SE on the TN substrates. Meanwhile, the morphological changes of the TN materials during SE processes were also analyzed. Enzymatic digestibility performance of raw and pretreated TN was compared to examine the effects of SE on saccharification efficiency. These findings from the present research would shed some light on the mechanisms of SE pretreatment and promote the valorization of TN biomass.

## Materials and methods

### Lignocellulosic materials and chemicals

The TN used in this study was cultivated in an experimental field of Inner Mongolia Academy of Agricultural & Animal Husbandry Sciences, China, and was harvested in September 2021. The overground tubers were collected, washed to remove impurity, and air-dried to constant weight. Cellulase cocktail (*Trichoderma viride* G) with average activity of 90.3 filter paper unit per gram was provided by Shanghai Yuanye Co., Ltd. Other chemicals (analytic grade) were purchased from Huhhot Shengkang Biotechnology Company, China.

### Steam explosion pretreatment

The dried overground tubers of TN was chopped to smaller sizes (~10 cm in length) and pre-soaked in water with a solid-liquid ratio of 1:10 (w/v) overnight to guarantee the saturation moisture. The saturated sample was drained off and placed into the SE equipment (Hebi Company, China) which consisted of a steam generator, a 400 mL reaction cylinder, and a collecting tank. As shown in Table 1, four conditions with different severity factor (SF) were selected to obtain various pretreated TN (PTN). SF of each condition was determined according to equation developed by Melissa (14), shown below:


(1) $$\begin{array}{l} SF\;=\;log_{10}\;\left[t*\exp\;\left(\frac{T-100}{14.75}\right)\right]\; \end{array}$$

**Table 1:** Conditions for SE pretreatment.

**Pressure (MPa)**	**Temperature (°C)**	**Time (min)**	**Severity factor (SF)**
1.2	188	5	3.29
1.5	198	10	3.89
1.8	207	10	4.15
1.5	198	20	4.19

Where *t* is the residence time, min; and *T* is the holding temperature, °C.

After each pretreatment, the PTN was washed in a water bath with a solid-liquid ratio of 1:10 (w/v) at room temperature for 30 min to remove the soluble compounds and then oven-dried at 63°C to a constant weight. The other saturated sample without a SE pretreatment was also oven-dried at 63°C to a constant weight and named as unpretreated TN (UTN). The PTN and UTN were shredded to powders with a particle size of 0.42–0.88 mm. Three repetitions were performed for each treatment.

### Chemical analysis

#### Composition analysis

The lignin and monomeric sugars in solid samples were analyzed using two-stage acid hydrolysis method recommended by National Renewable Energy Laboratory (15). The concentration of monosaccharides were analyzed through HPLC system equipped with a Bio-Rad Aminex HPX-87H column, with 5 mM sulfuric acid as mobile phase, eluted flow rate of 0.6 mL/min, and column temperature of 50°C.

#### Fourier transform infrared spectroscopy

The Infrared spectra of PTN and UTN in the region 4,000–400 cm^−1^ were obtained by FTIR spectrophotometer (IR-Affinity-1S, Shimadzu) in transmission mode, with 4 cm^−1^ resolution and 32 scans at room temperature. Milled sample powder (1 mg, 60 mesh) was mixed with of KBr (50 mg, spectrum grade) and pressed into a pellet. The characteristic bands were identified and analyzed according to literatures (16).

### Physical analysis

#### Cellulose accessibility

The cellulose accessibility of various solid substrates was determined with the theoretical maximum adsorption amount of Congo Red (17). Precise 200 mg of dry sample was incubated with 4 mL of dye sodium citrate buffer (50 mM, pH 4.8) in a 10 mL centrifuge with gentle agitation at 50°C for 24 h. After centrifugation at 6,200 × g for 10 min, the absorbance of the supernatant was measured using UV-visible spectrophotometer (UV-1780, Shimadzu, Japan) at 498 nm. The adsorption isotherm was determined in duplicate with a series of increasing dye concentrations (0, 0.1, 0.5, 2.0, 3.0, and 4.0 g/L) and fitted by linear regression in Excel.

#### X-ray diffraction

The XRD patterns of various samples were detected on a X-ray diffractometer instrument (X'Pert PRO, PANalytical) using Cu *K*_α_ radiation (*k* = 1.54 Å), with a scattering angle (2θ) ranging from 5° to 40° and scanning increments of 0.02°, at 40 kV and 30 mA. The crystallinity index (CrI) was calculated as follows (18).


(2) $$\begin{array}{l} CrI\;=\;\left(I_{002}-I_{am}\right){\;}^{*}\;100/I_{002} \end{array}$$

Where *I*_002_ represents the peak intensity of the crystalline area of the biomass in approximate 2θ between 22° and 23°; *I*_*am*_ represents the minimum intensity of the amorphous area in ~*2*θ between 18° and 19°.

### Microscope imaging analysis

Transverse sections (12 μm) of various samples were obtained by cryostat (Leica, RM2015) at −20°C. Whole and uniform sections were maintained on a glass slide and observed by confocal laser scanning microscopy (CLSM, NIKON, AIR) with the identical condition: 20 × /1.40 NA Plan-Apochromatic objective lens, laser source at 20% power, and pinhole size of 1 AU. Excitation was conducted using a wavelength of 405 nm for lignin autofluorescence, and emissions 410–480 nm (blue fluorescence) was collected. At least five images for each sample were collected.

### Enzymatic hydrolysis

Enzymatic hydrolysis was carried out with a cellulase loading of 20 FPU/g in 100 mL Erlenmeyer flasks containing 2 g of solid substrate and 40 ml of sodium citrate buffer (50 mM, pH 4.8, supplementing with 0.01 g/mL sodium azide) in a water–bath. The Erlenmeyer flasks were incubated in a shaker at 50°C and shaken at 150 rpm for 48 h. The generated glucose in enzymatic hydrolysates was analyzed on HPLC. The cellulose conversion based on cellulase mediated hydrolysis was used to express the enzymatic digestibility, which is calculated as the following Equation (19).


(3) $$\begin{array}{l} Cellulose\;conversion\;=\;\left({m_{g}}^{*}0.9+{m_{c}}^{*}0.95\right)/m \end{array}$$

Where *m_g_* and *m_c_* represents the amount of generated glucose and cellobiose during the enzymatic hydrolysis, respectively; 0.9 and 0.95 represents the conversion factor for glucan to glucose and cellobiose to glucose, respectively; *m* represents the amount of cellulose in substrate.

## Results and discussion

### Chemical properties of raw and pretreated tiger nut

#### Chemical composition

**Figure 1:**
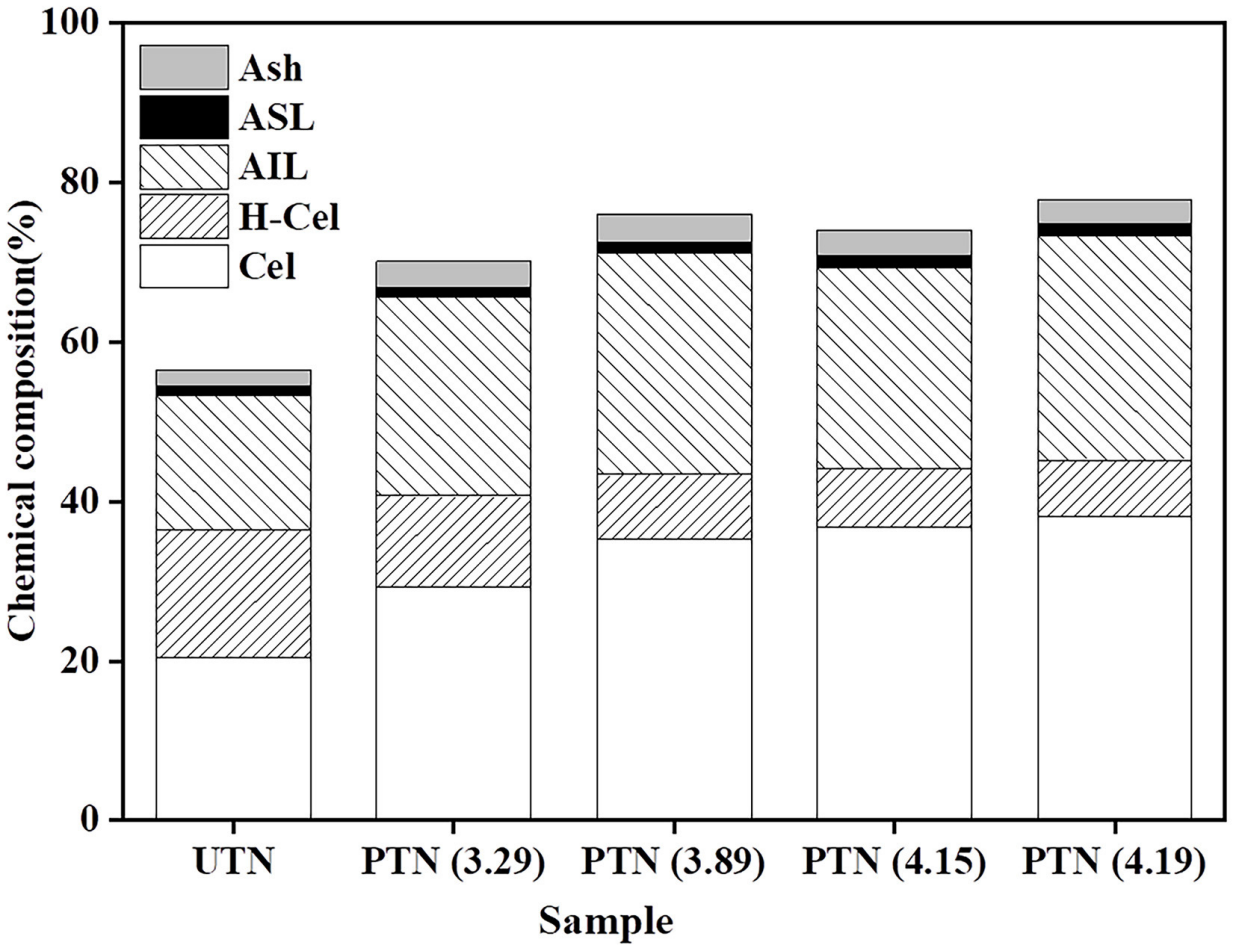
Compositions of TN before and after SE pretreatment.

Chemical composition is of great interest as it relates to the pretreatment effectiveness and further conversion performance (20). It was found that the glucan content increased from 20.51% (UTN) to 38.22% (SF = 4.19) with the increment of SF (Figure 1), which was beneficial for increasing the cellulose accessibility and enhancing the enzymatic hydrolysis performance. In contrast, the xylan content gradually decreased from 15.96 to 6.97%, and the declining trend was not obvious when the SF reached 4.15. The results showed that SE removed hemicellulose significantly from the solid biomass materials. The xylan content decreased from 14.51 to 3.51% in the solid substrates as the SE severity increased. Eom and coworkers also reported that SE pretreatment degraded hemicellulose within rubber wood biomass, which was in line with the results in the present study (21). Lignin generally acts as a physical barrier or irreversibly adsorbs cellulases to prevent cellulose hydrolysis (22). However, inconsistent trend of lignin content during SE is often reported. Most published literatures reported that lignin content increased in the SE treated biomass substrates. For example, Nasir et al. found that the lignin content increased by 25.85% in the SE (1.0 MPa, 184°C, 15 min) treated corn stover (23). Whereas, the other party assumed the decreased results of lignin contents during the SE. Besserer and coworkers reported a delignification of 3 to 75% with an average of ~25% after SE treatments on *Aucoumea klaineana Pierre* sapwood based on both the microscopical data and chemical analysis (24). The lignin profiles after SE pretreatment was investigated in the present study to reveal the effects of SE on TN lignin. Results showed that the lignin content within PTN increased after SE pretreatment. It was deduced that lignin content variation might depend on the SE pretreatment time and temperature. Overall, SE pretreatment altered the compositions of TN materials, which should influence the subsequent enzymatic digestibility performance.

#### FTIR spectra

**Figure 2:**
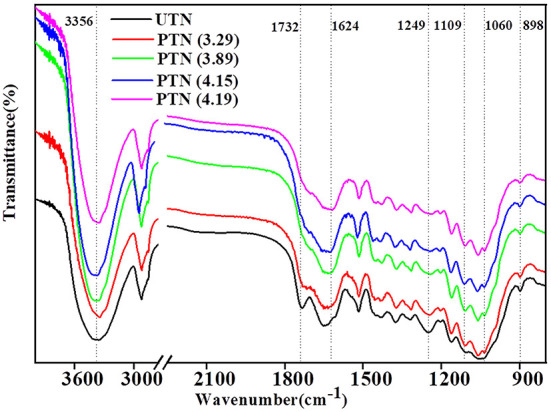
FTIR spectra of TN before and after SE pretreatment.

The FTIR spectra in band shapes of UTN and PTN samples were presented and compared to clarify the details of chemical variations, especially for the representative spectral region of 1,800–800 cm^−1^. As shown in Figure 2, there was a significant peak at 3,356 cm^−1^ (O–H bending in polysaccharides) in all samples, indicating the preservation of cellulose. The mild decrease in the band at 898 cm^−1^ (β-glucosides in cellulose) after SE treatments suggested the breakage of β-1,4 glycosidic bonds in cellulose. Our previous study found that SE could depolymerize cellulose in the wheat bran to release glucose, which benefited to the subsequent biological conversion (25). The disappearance of the band near 1,732 cm^−1^ (C=O stretching in hemicellulose) and 1,249 cm^−1^ (C–O stretching hemicellulose and syringyl ring in lignin) implied the degradation of hemicelluloses and breakage of lignin and hemicellulose, which was in line with the results of hemicellulose content decrease. Slight and continuous increase in the band near 1,109 cm^−1^ (C–OH skeletal vibration) and 1,060 cm^−1^ (C–O deformation vibration in secondary alcohols or aliphatic ethers) suggested the chemical modification of lignin. Maniet and coworkers systematically investigated the influences of SE on lignin structure and revealed that an increase of the SE intensity induced β-O-4 and spirodienone substructure degradations, increase in COOH content and phenolic OH bonds, decrease of aliphatic OH ferulate and *p*-coumarate bonds, and changes in subunits proportions within the organosolv fescue lignin (26). Lignin degradation is an essential step for its bioconversion. Therefore, the SE treated lignin might be suitable for biological valorization for producing value-added products (e.g., lipids and polyhydroxyalkanoates). As the degradation of hemicellulose and lignin was observed, the inhibiting compounds should be generated during the SE treatment. Fortunately, these inhibitors can be removed effectively by facile detoxification methods (e.g., water-washing and drying). Li and Chen reported that water-washing removed 81% of the furfural and 85% of the phenol compounds from SE treated corn straw and hot air-drying can obtained 46% of the furfural removal and 8.1% of the phenol compounds removal, respectively (27). In the present study, the PTN samples were washed in a water bath with a solid-liquid ratio of 1:10 (w/v) at room temperature for 30 min and then oven-dried at 63°C to a constant weight to eliminate the influence of the inhibiting compounds on the following biological valorization processes. Overall, SE removed hemicellulose and broke the linkages of lignin, which mitigated the recalcitrance of the TN biomass to cellulases.

### Physical properties of raw and pretreated tiger nut

**Figure 3:**
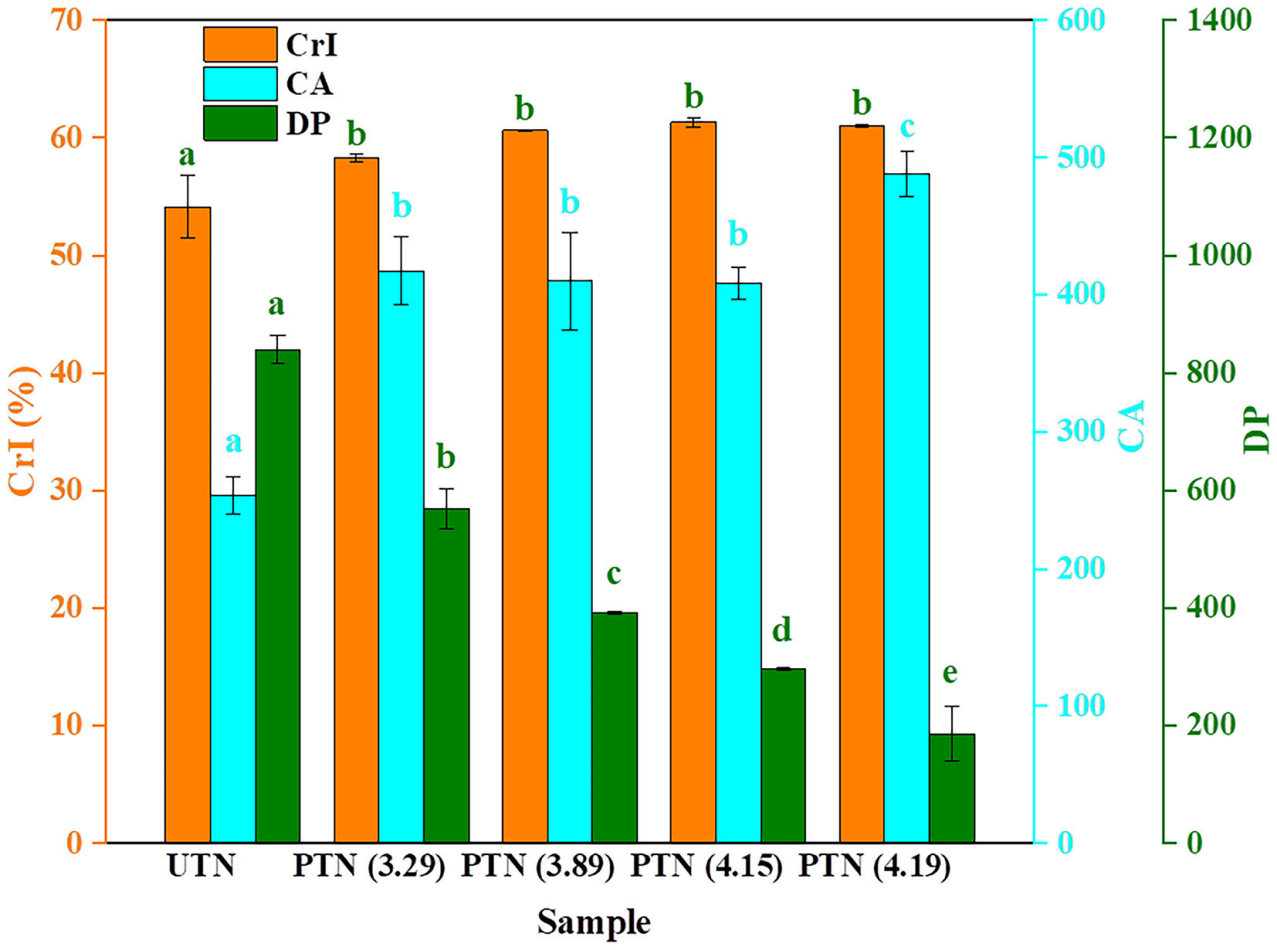
Physical properties of TN before and after SE pretreatment. Bars followed by the different superscripts (a–e) show significant difference (*p* < 0.05) according to the Duncan's multiple range test.

Cellulose consists of crystalline and amorphous component. Additionally, the lignin and hemicellulose were deemed as amorphous components. XRD patterns depicted typical cellulose Iβ allomorphs in the samples regardless of SE pretreatments. The CrI of UTN (54.15%) was lower than that of PTN samples which ranged from 58.25 to 61.33% (Figure 3). This result revealed the disintegration of the amorphous components (e.g., hemicellulose, lignin, and extractives) by the SE treatments. Ying et al. also found that the degradation of hemicellulose in poplar by an acetic acid hydrolysis pretreatment resulted in an increase of the CrI value, which is in accordance with the present work (28). No significant increase in CrI of PTN was observed as the SF increased from 3.29 to 4.19. This indicated that the crystalline component became correspondingly more exposed despite of little disruption of hydrogen bonds within cellulose, which was responsible for the cellulase adsorption. Cellulose accessibility (CA) correlating to the adsorption behavior of cellulase directly impacts the sugar release. CA increased from 253.53 in UTN to 488.10 of PTN (SF = 4.19) as the severity of SE pretreatment increased, which can be primarily attributed to that the SE allows hemicelluloses solubilization and opens the lignocellulosic structure (26, 29). Thus, SE pretreatment enhanced the accessibility of the cellulose, which could subsequently promote the enzymatic digestibility. An obvious decrease of degree of polymerization (DP) was observed, in detail, the DP decreased from 839.85 (UTN) to 186.08 (SF = 4.19), which represented a 77.84% reduction of cellulose DP. This result suggested the breakage of inner linkages within cellulose chains during the SE process. This was aligned with the above hypothesis that SE could not only degrade the amorphous component but also destroyed basic bonds in cellulose, which resulted in the depolymerization and exposure of the cellulose (30). Huang and coworkers also reported a decrease of cotton stalk cellulose DP by 50–65% after SE (SF = 4.22). Furthermore, they found that the reduction of DP was one of the main factors enhancing the enzymatic hydrolysis (31). Overall, these physical parameter changes of TN suggested that more accessible substrates for enzymatic hydrolysis were presented by the SE pretreatment.

### Morphological properties of raw and pretreated tiger nut

**Figure 4:**
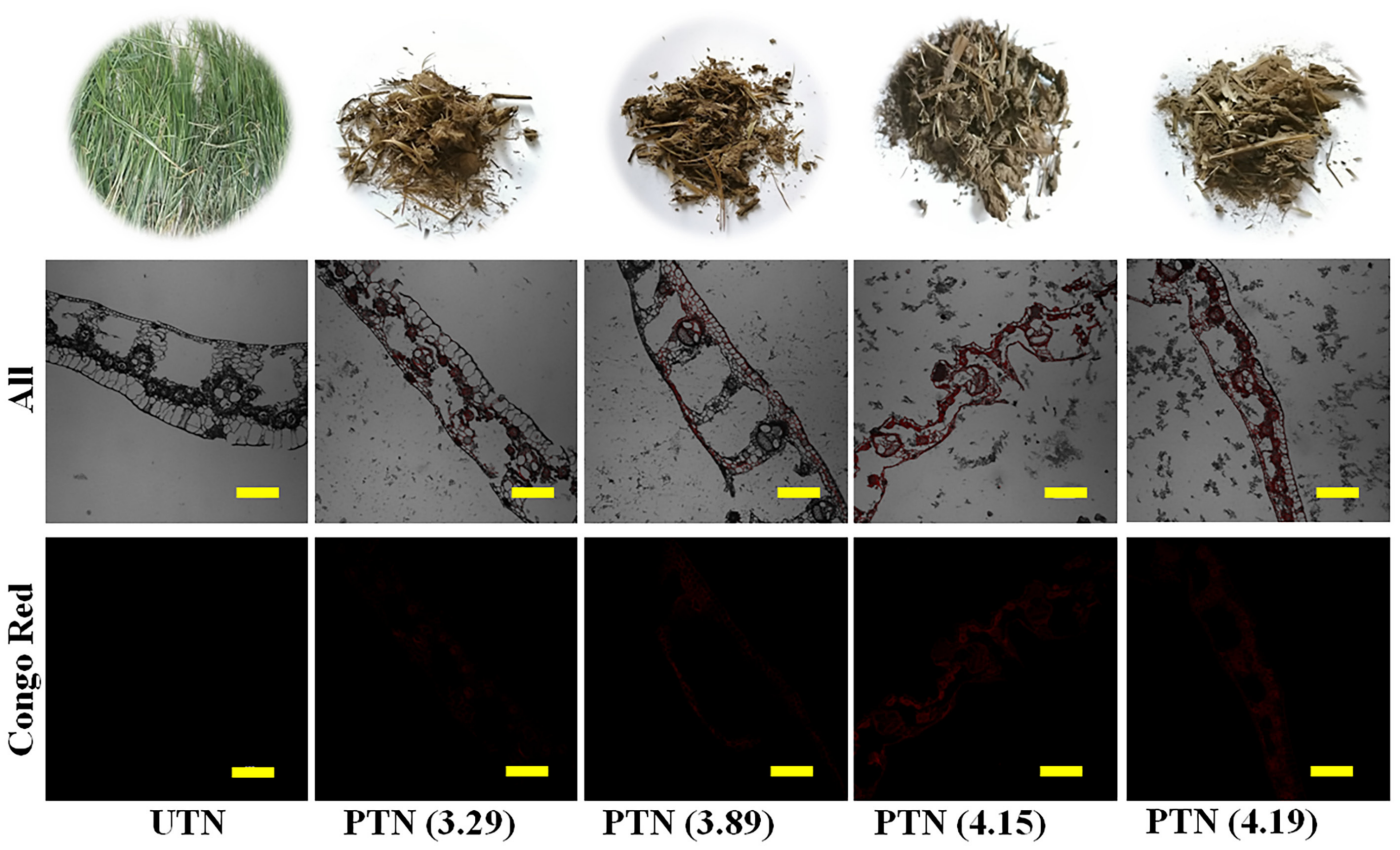
Photos (top) and CLSM images of TN before and after SE pretreatment. Red was cellulose labeled with Congo. Scale bar = 100 μm.

Morphological alterations of TN during the SE pretreatments were observed using the CLSM imaging techniques. Visual observation suggested that the PTN samples were darker compared to UTN and slightly agglomerated but readily divided (Figure 4). Also, the UTN had rigid and smooth fibers, whereas the PTN looked porous, rough, irregular, and defibrillated. Color variation is a primary indicator of chemical variations in the lignocellulosic biomass. Similar results were also obtained from the SO_2_-catalyzed steam treated softwood pellets (32). The enhanced darkness was associated with surface burning and dissolution of extractives, lignin, and hemicellulose, which resulted in the destruction in mechanical strength and dimensional stability, providing readily degraded substrates for enzymes (33).

Based on the microscopic inspection, it was found that SE pretreatment destroyed the rigid structure and dislocated the cell wall. Specially, dramatic structure changes were observed after SE pretreatment with a higher severity. In detail, during the high temperature cooking stage of the SE, the high temperature steam softened the TN biomass matrix structure. Meanwhile, hemicellulose was hydrolyzed by acetic acid derived from acetyl groups and/or other acids. Some inner linkages of lignin were destroyed and cellulose binding was reduced (34). During the subsequent instantaneous decompression stage, the structure of TN substrates was destroyed by the combined effects of water flashing and volume expansion. Furthermore, the TN biomass materials knocked against the inner-wall of the instrument and collided each other, resulting in a destroyed matrix structure of the biomass (30). As shown in Figure 4, when SF reached 4.15 or larger, the TN matrix was disrupted significantly. Therefore, SE treatment reduced the recalcitrance and presented a easily accessible TN substrate for the enzyme attack.

Furthermore, The CLSM images showed that almost no Congo Red was absorbed by the UTN. This phenomenon demonstrated that the cellulose was difficult to access in the raw TN materials, which also confirmed the recalcitrance in the natural plants (35). With the SF increase, it is interesting to note that the signal intensity of Congo Red became stronger. This result suggested that more cellulose were exposed as the SE severity increased. Accordingly, the cellulose could be more accessible by the cellulase, which was in line with the result that CA increased with the SF increase. The CLSM imaging techniques provided a more visible observation and easy understanding regarding the cellulose expose by the SE treatments (36). Therefore, SE pretreatment destructed the rigid structure and exposed cellulose in TN materials, which could facilitate the subsequent saccharification.

### Enzymatic digestibility performance of raw and pretreated tiger nut

**Figure 5:**
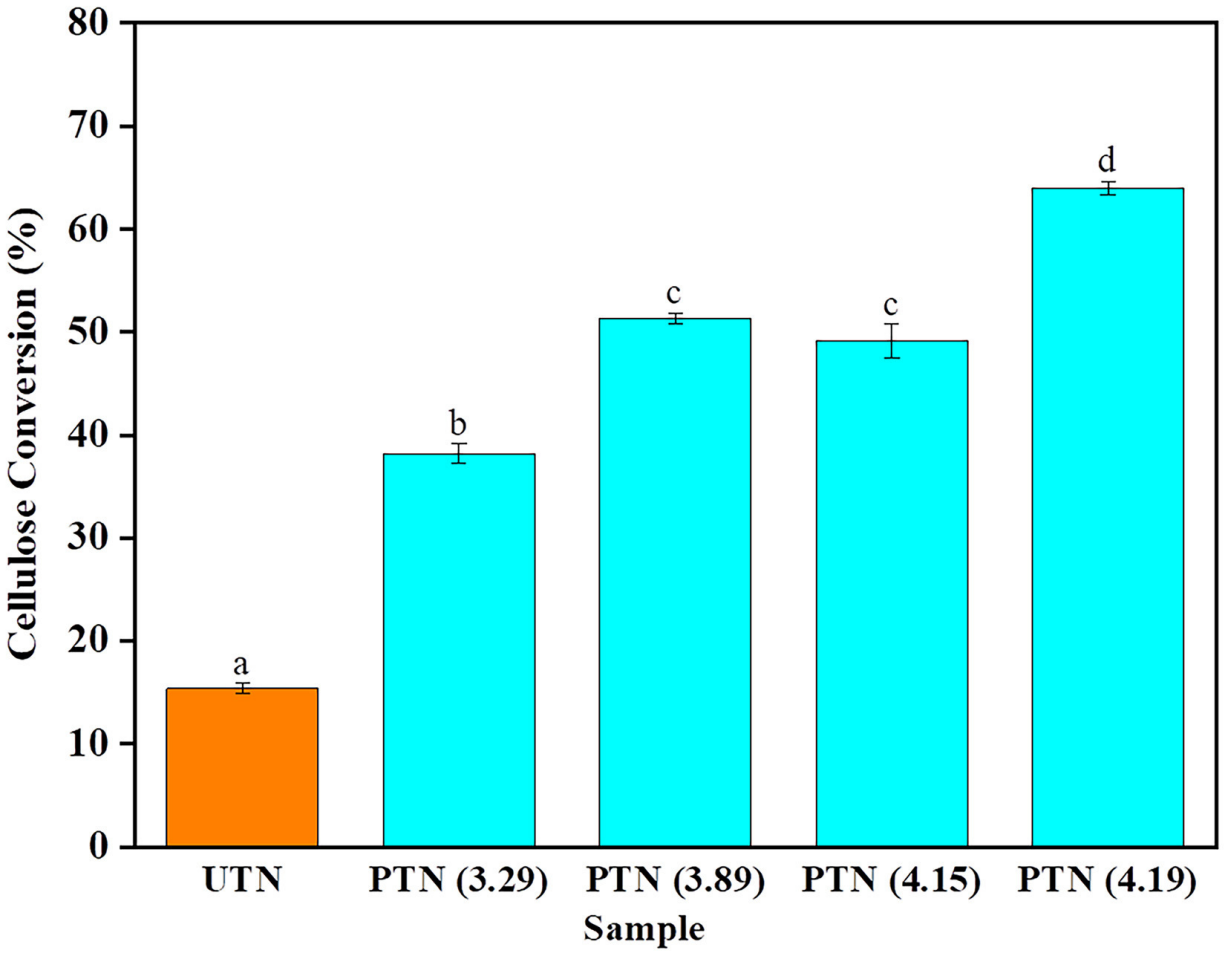
Cellulose enzymatic hydrolysis performance of TN before and after SE pretreatment. Bars followed by the different superscripts show significant difference (*p* < 0.05) according to the Duncan's multiple range test.

Cellulase was added to the TN substrates regardless of SE pretreatments to release fermentable sugars that can be further used for biofuels production. Previous study reported that a cellulase loading of 20 FPU/g was beneficial to the enzymatic hydrolysis (37). To investigate the effects of SE pretreatment on saccharification of TN biomass, enzymatic hydrolysis was conducted for 48 h with an enzyme loading of 20 FPU/g. As shown in Figure 5, the cellulose conversion of UTN was merely 15.39%. With the SF increased during the SE pretreatment, the cellulose conversion of PTN ranged from 38.18 to 63.97%, which was 2.5–4.2 times larger than that of the UTN. These results implied that SE significantly improved the cellulose conversion efficiency. Interestingly, the difference of cellulose conversion between PTN with SF of 3.89 (1.5 MPa, 10 min) and PTN with SF of 4.15 (1.8 MPa, 10 min) was not significant. Whereas, the cellulose conversion of PTN with SF of 4.19 (1.5 MPa, 20 min) reached 63.97%. These results suggested that the longer boiling time presented more significant impact on enzymatic digestibility than the higher boiling temperature during the SE process. Overall, SE broke the linkages of hemicellulose and lignin and increased the cellulose content within the solid TN substrates. Meanwhile, SE disrupted inner linkages within cellulose chains, reducing the degree of polymerization. As a result, cellulose after SE treatment was more easily accessible by the enzymes (38). Also, the smaller cellulose molecules were more readily degraded, resulting in a significantly improved saccharification efficiency. Enzymatic hydrolysis of cellulose to release fermentable sugars is a prerequisite determining the bioethanol production. Enzymatic digestibility is a significant indicator to evaluate the biorefinery performance of plant materials. For example, Liu et al. examined the enzyme digestibility of corn stover with different particle size to choose the optimal particle size for corn stover biomass utilization. It was found that the utilization of larger biomass particles was desirable for biofuels production and reduced process cost (39). In the present study, the enhanced enzymatic digestibility performance of the steam explosion treated TN demonstrated great potential for the value-added utilization of TN for a biorefinery scenario.

## Conclusion

TN grows well in well-drained sandy or loamy soils. The overground tubers of TN represent promising resources for biorefineries. By applying a steam explosion pretreatment, physical-chemical properties of the overground tubers of TN was significantly improved for facilitating the enzymatic hydrolysis to release fermentable sugars. In particular, SE increased cellulose content through removing the hemicellulose from the solid biomass. Moreover, SE reduced the degree of polymerization and improved the accessibility of cellulose, which benefited to the enzymatic adsorption and digestibility. The saccharification efficiency of the TN substrates was significantly enhanced by the SE pretreatment. Therefore, SE is an effective method for promoting the valorization of TN biomass toward a sustainable bioenergy production.

## Data availability statement

The original contributions presented in the study are included in the article/supplementary material, further inquiries can be directed to the corresponding authors.

## Author contributions

Z-MZ: writing and editing. WY: methodology, investigation, and formal analysis. CH: investigation and data curation. JL: investigation. HX: review and editing. DZ: review, editing, and funding acquisition. GL: conceptualization, design, and writing. All authors contributed to the article and approved the submitted version.
